# Effects of Reorientation of Graphene Platelets (GPLs) on Young’s Modulus of Polymer Composites under Bi-Axial Stretching

**DOI:** 10.3390/nano8010027

**Published:** 2018-01-07

**Authors:** Chuang Feng, Yu Wang, Jie Yang

**Affiliations:** School of Engineering, RMIT University, P.O. Box 71, Bundoora, VIC 3083, Australia; chuang.feng@rmit.edu.au (C.F.); s3415279@student.rmit.edu.au (Y.W.)

**Keywords:** micromechanics, graphene platelets, nanocomposites, bi-axial stretching, orientation distribution

## Abstract

Effects of bi-axial stretching induced reorientation of graphene platelets (GPLs) on the Young’s modulus of GPL/polymer composites is studied by Mori-Tanaka micromechanics model. The dispersion state of the GPLs in polymer matrix is captured by an orientation distribution function (ODF), in which two Euler angles are used to identify the orientation of the GPLs. Compared to uni-axial stretching, the increase of the stretching strain in the second direction enhances the re-alignment of GPL fillers in this direction while it deteriorates the re-alignment of the fillers in the other two directions. Comprehensive parametric study on the effects of the out-of-plane Young’s modulus, stretching strain, strain ratio, Poisson’s ratio and weight fraction and GPL dimension on the effective Young’s moduli of the composites in the three directions are conducted. It is found that the out-of-plane Young’s modulus has limited effects on the overall Young’s modulus of the composites. The second stretching enhances the Young’s modulus in this direction while it decreases the Young’s modulus in the other two directions. The results demonstrate the increase of Poisson’s ratio is favorable in increasing the Young’s modulus of the composites. GPLs with larger diameter-to-thickness ratio have better reinforcing effect on the Young’s modulus of GPL/polymer nanocomposites.

## 1. Introduction

Recently, adding graphene and its derivatives to polymers as reinforcements to produce high performance composites and structures has stimulated a surge of scientific interest from various engineering fields [1,2,3,4,5,6]. Such interests stem from graphene and its derivatives’ excellent mechanical and physical properties and improved reinforcing effects compared to other carbon-based fillers, such as carbon fibers (CFs) and carbon nanotubes (CNTs). For example, the in-plane Young’s modulus and tensile strength of graphene are measured to be as high as 1 TPa and 130 GPa, respectively [7]. With the same reinforcement loading, i.e., 0.1% weight fraction (w.t.), Rafiee et al. [8] found the Young’s modulus of graphene platelet (GPL) reinforced epoxy composites was increased by 31%, but only a 3% increases was achieved by using CNT fillers. Liang et al. [9] also demonstrated that adding 0.7 w.t. % graphene oxide (GO) into poly(vinyl alcohol) increased the tensile strength and Young’s modulus by 76% and 62%, respectively. It was experimentally evidenced by Lee et al. [10] and Shokrieh et al. [11] that the addition of graphene into epoxy could significantly enhance the strength and toughness of the composites. Recently, Liu et al.’s [12] experiments showed that the addition of GPL could significantly improve the tensile and dynamic mechanical properties of Poly(methyl methacrylate) (PMMA) nanocomposites. Their examination on GPL thickness effects found that better mechanical properties of the nanocomposites could be achieved by using GPLs with thinner thickness. It is also found that the dispersion of graphene in polymer can significantly influence the properties of the graphene reinforced polymer composites. For example, Tang et al. [13] studied the effects of dispersion of graphene on the properties of polymer composites. It was observed that highly dispersed graphene produced better mechanical and electrical properties of the composites than the poorly dispersed case. Kim et al. [14] found that different processing routes to prepare graphene/polymer composites could have difference effects on the electrical conductivity of the composites due to the different dispersion level of graphene in the polymer matrix. In addition to experiments, extensive theoretical investigations, including molecular dynamics (MD) simulation [15,16,17,18], micromechanics modelling [19,20] and finite element method (FEM) [21,22,23,24,25], also observed the significant reinforcing effects of graphene and its derivatives. The mechanisms that underpin the prominent reinforcing effects can be attributed to graphene and its derivatives’ 2D structure features and their extremely high surface area, which result in excellent load transfer from the matrix to the reinforcements [1,8,11,20,26,27]. Moreover, the abundance of graphite in nature can significantly reduce the cost of some graphene’s derivatives, such as GO and GPL, which enables their applications in large scale engineering structures possible and practical.

An understanding on the mechanical properties of graphene-based polymer composites is of great importance for the material design and engineering application. Although lots of efforts have been devoted to investigating the mechanical properties of the composites as mentioned above, the majority of previous studies are focused on preparing composites with well-dispersed graphene fillers or predicting the overall mechanical properties of the composites without considering the effects of fillers’ orientation and distribution. However, it has been experimentally demonstrated that the mechanical properties of the composites are highly dependent on the orientation of the reinforcing fillers. For example, exposing CNT reinforced composites to a magnetic field, Camponeschi et al. [28] found the Young’s modulus of the composites was considerably improved due to the re-alignment of CNTs. Using Raman spectroscopy, Li et al.’s [29] experiments showed the Young’s modulus of CNT reinforced composites was much smaller than that of the composites with perfect alignment of CNTs in the longitudinal direction. Deniz Ürk et al. [30] obtained the same conclusion by doing dynamic analysis on CNT reinforced composites. More work on the effects of nanofillers’ orientation and distribution on mechanical properties of composites can be found in [31,32,33,34,35,36]. In addition to applying electrical field [37,38,39] and magnetic field [28,40,41], mechanical stretching [42,43,44,45] is also recognized as a simple but effective way to re-orientate nanofillers in composites. An investigation on the effects of mechanical stretching induced filler’s re-orientation will offer suggestions on tailoring the mechanical properties of the composites. Recently, Feng et al. [46] studied the uni-axial stretching induced re-orientation effects on Young’s modulus. Unfortunately, to the best of the authors’ knowledge, limited theoretical work has been done to quantify the effects of bi-axial stretching, which is recognized as a typical stretching mode for re-orientation of reinforcing fillers [47,48], induced re-orientation of fillers on the mechanical properties of the composites. 

This paper investigates the effects of bi-axial stretching induced re-orientation of GPLs on the Young’s modulus of the GPL/polymer composites by employing Mori-Tanaka micromechanics model. The re-orientation of GPLs is captured through an orientation distribution function (ODF), in which the variations of two Euler angles, identifying filler’s orientation, are considered. To simplify the modelling, the micromechanics model in present work assumes perfect bonding between the two phases of the composite, i.e., GPLs and the polymer matrix, when subjected to small deformation. The interfacial effects caused by the interaction between graphene and the polymer matrix are also neglected in present paper. However, it should be noted that as evidenced by Skountzos et al. [18] such interfacial effects could have significant impact on the mechanical properties of the composites. Moreover, when the composites are subjected to large deformation, debonding/slipping between the fillers and the matrix may become significant. Under such circumstances, a cohesive law may be needed in the micromechanics model to capture the above mentioned limitations. 

## 2. Effective Mechanical Properties of GPL/Polymer Composites

**Figure 1:**
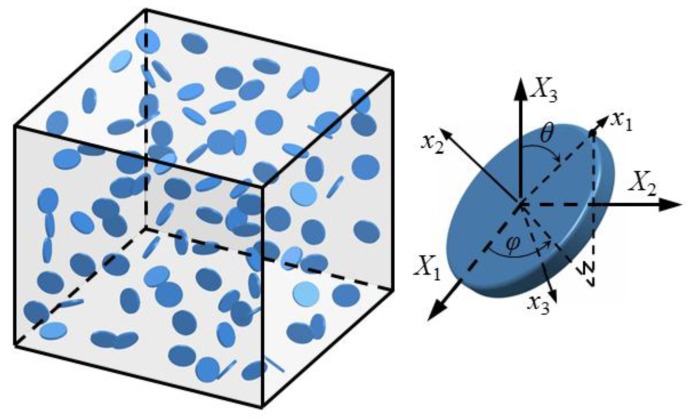
Microscale RVE and characterization of GPL orientation.

Assuming GPLs as flat disks [31,49,50] dispersed in polymer matrix, the mechanical properties of this composite can be studied by choosing a representative volume element (RVE), containing enough fillers distributed in any possible orientations (as shown in Figure 1). To identify the GPL in the RVE, three Euler angles are normally required to depict the orientation of the fillers in three dimensional geometry. Since perfect bonding between the reinforcements and the polymer is assumed, the GPLs in the polymer matrix will not be capable of moving freely when the composites are subjected to bi-axial stretching. Under such circumstances, the orientation of each individual GPL disk in this RVE can be characterized by two Euler angles, i.e., polar angle θ and azimuth angle ϕ. When the GPL disks, with diameter and thickness being *d*_GPL_ and *t*_GPL_, respectively, are randomly and uniformly distributed in the RVE before applying stretching. The overall mechanical properties of this two phase composites can be homogenized and approximated by a micromechanics model. In present work, Mori-Tanaka method is adopted as the micromechanics model for the GPL reinforced polymer composites. As a typical mean field approximation method, Mori-Tanaka model is based on Eshelby’s elasticity solution for the inhomogeneities, i.e., GPL fillers. It uses concentration tensors to correlated average state variables, i.e., stress or strain, in inhomogeneities and polymer matrix to the average macroscopic variables of the composites. Compared to other micromechanics model, Mori-Tanaka micromechanics model is able to take the interaction among inhomogeneities into account. Using Mori-Tanaka model, the overall mechanical properties of the two-phase GPL/polymer composite can be obtained by averaging the contribution of the fillers from all possible orientations [51,52,53] as shown in Figure 1.

(1) $$C_{\mathrm{eff}}=C_{M}+V_{\mathrm{GPL}}\langle \left({\tilde{C}}_{\mathrm{GPL}}-C_{M}\right){\tilde{A}}_{\mathrm{dil}}\rangle {\left[\left(1-V_{\mathrm{GPL}}\right)I+V_{\mathrm{GPL}}\langle {\tilde{A}}_{\mathrm{dil}}\rangle \right]}^{-1}$$

where ***C***_eff_ and ***C***_M_ are the effective elastic stiffness tensors of the composite and the polymer matrix in the global coordinate system (*X*_1_, *X*_2_, *X*_3_) of the RVE, respectively, ${\tilde{C}}_{\mathrm{GPL}}$ is the stiffness tensor of the GPL in the local coordinate system (*x*_1_, *x*_2_, *x*_3_), *V*_GPL_ is the volume fraction of the GPL, and the angle bracket ‹ › represents term averaging over all orientations in the global coordinate system. Here the stiffness tensor in present work does not consider the tangent stiffness to simplify the model. Then the stiffness tensors for polymer matrix and the GPL in the composite are reduced to ***C***_M_ = *E*_M_**I** and ${\tilde{C}}_{\mathrm{GPL}}=\left(E_{\mathrm{in}},E_{\mathrm{ou}},E_{\mathrm{in}}\right)I$, respectively, where *E*_M_ is the Young’s modulus of the polymer matrix and *E*_in_ and *E*_ou_ are the in-plane and out-of-plane Young’s moduli of the GPL in the local coordinate system, respectively. ${\tilde{A}}_{\mathrm{dil}}$ in Equation (1) is the dilute mechanical strain concentration tensor for the filler, i.e.,

(2) $${\tilde{A}}_{\mathrm{dil}}={\left\{I+\tilde{S}{\left({\tilde{C}}_{M}\right)}^{-1}\left({\tilde{C}}_{\mathrm{GPL}}-C_{M}\right)\right\}}^{-1}$$

***S*** is the Eshelby tensor for disk shape filler written as [51]

(3) $$\tilde{S}=\left[\begin{array}{ccc} S_{11} & 0 & 0\\ 0 & S_{22} & 0\\ 0 & 0 & S_{33} \end{array}\right]$$

where $S_{11}=S_{33}=\frac{\pi t_{\mathrm{GPL}}}{4d_{\mathrm{GPL}}}$ and $S_{22}=\frac{\pi t_{\mathrm{GPL}}}{2d_{\mathrm{GPL}}}$, respectively. Accounting for all possible orientations of the fillers in the composite, the average of dilute concentration tensor can be written as [52,54]

(4) $$\langle \tilde{A}\rangle =\frac{\int_{0}^{2\pi }\int_{0}^{\pi }\tilde{A}\rho \left(\varphi ,\theta \right)\sin\theta d\theta d\varphi }{\int_{0}^{2\pi }\int_{0}^{\pi }\rho \left(\varphi ,\theta \right)\sin\theta d\theta d\varphi }$$

where *ρ*(ϕ,θ) is the orientation distribution function (ODF) describing the probability density of GPL distribution for a given set of Euler angles. When GPLs are randomly and uniformly dispersed in polymers before stretching, each orientation will have the same probability density and the ODF is equal to unity, i.e., *ρ*(ϕ,θ) = 1. However, when subjected to deformation, fillers in the composites will be re-orientated due to the transferred shear force from the polymer matrix. Under such circumstance, the ODF becomes dependent on the distribution of the fillers. This will lead to the variation of the mechanical properties of the composites. 

The concentration tensor of the filler in the local coordinate system (*x*_1_, *x*_2_, *x*_3_) can be transformed into the one in the global coordinate system as [51,55,56]

(5) $$A=Q^{T}{\tilde{A}}^{\mathrm{dil}}Q{\left\{\left(1-V_{\mathrm{GPL}}\right)I+\frac{V_{\mathrm{GPL}}}{4\pi }\int_{0}^{2\pi }\int_{0}^{\pi }\left\{Q^{T}{\tilde{A}}^{\mathrm{dil}}Q\right\}\sin\theta d\theta d\varphi \right\}}^{-1}$$

where *Q* is the transformation matrix in terms of the two Euler angles written as [54]

(6) $$Q=\left[\begin{array}{ccc} \sin\theta \cos\varphi & -\cos\theta \cos\varphi & \sin\varphi \\ \sin\theta \sin\varphi & -\cos\theta \sin\varphi & -\cos\varphi \\ \cos\theta & \sin\theta & 0 \end{array}\right]$$

Combining Equations (1)–(5), the overall elastic stiffness tensor for the GPL/polymer composites becomes

(7) $$C_{\mathrm{eff}}=C_{M}+\frac{\int_{0}^{2\pi }\int_{0}^{\pi }\rho \left(\varphi ,\theta \right)V_{\mathrm{GPL}}\left(C_{\mathrm{GPL}}-C_{M}\right)A\sin\theta d\theta d\varphi }{\int_{0}^{2\pi }\int_{0}^{\pi }\rho \left(\varphi ,\theta \right)\sin\theta d\theta d\varphi }$$

where $C_{\mathrm{GPL}}=Q^{T}{\tilde{C}}_{\mathrm{GPL}}Q$ denotes the stiffness tensor of the GPL in the global coordinate system. 

## 3. Orientation Distribution Function (ODF)

**Figure 2:**
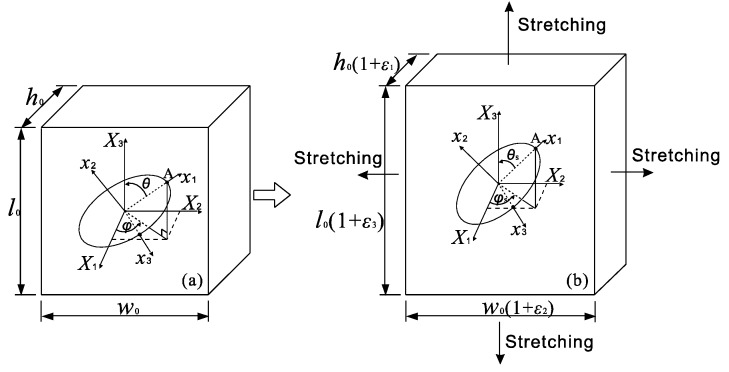
Re-orientation of GPL in a cell subjected to bi-axial stretching.

To determine the ODF for the fillers in the RVE after stretching, a unit cell containing one GPL disk (as shown in Figure 2) is considered. The three dimensions in *X*_1_, *X*_2_ and *X*_3_ directions are *h*_0_, *w*_0_ and *l*_0_, respectively. The original polar and azimuth angles of the filler are θ and ϕ, respectively. When this unit cell is subjected to a bi-axial stretching, i.e., ε_3_ and ε_2_ in X_3_ and X_2_ directions, respectively, the three dimensions of the cell become *l* = *l*_0_(1 + ε_3_), *ω* = *ω*_0_(1 + ε_2_) and *h* = *h*_0_(1 + ε_1_), respectively. In the following, ε_3_ and ε_2_ are defined as principle and the second stretching strains, respectively, which satisfy ε_3_ ≥ ε_2_. The resultant strain *ε*_1_ in *X*_1_ direction due to the bi-axial stretching can be determined by Equation (8),

(8) $${d\varepsilon }_{3}=-\frac{\nu }{1-\nu }\left({d\varepsilon }_{1}+{d\varepsilon }_{2}\right)$$

where *ν* is the Poisson’s ratio of the unit cell. Integrating this expression over the elongations in the three directions, i.e., *Δl*, *Δω* and *Δh*, the strain *ε*_1_ can be derived as [57]

(9) $$\varepsilon_{1}={\left[\left(1+\varepsilon_{3}\right)\left(1+\varepsilon_{2}\right)\right]}^{-\frac{\nu }{1-\nu }}-1$$

In particular, when ε_2_ = (1 + ε_3_)^−ν^ − 1, the bi-axial stretching reduces to uni-axial stretching case, i.e., ε_1_ = ε_2_ = (1+ ε_3_)^−ν^ − 1. Since the stiffness of the GPL is much higher than that of the polymer matrix, when subjected to stretching, the deformation of the composite is mainly accommodated by the polymer matrix while the GPL’s deformation, i.e., elongation, can be neglected. Under such circumstance, the effective volume fraction of the GPLs in the composite after the bi-axial stretching is updated as

(10) $$V_{\mathrm{update}}=\frac{V_{\mathrm{GPL}}}{{\left[\left(1+\varepsilon_{3}\right)\left(1+\varepsilon_{2}\right)\right]}^{\frac{1-2\nu }{1-\nu }}}$$

In addition to the deformation of the unit cell, the GPLs will be re-orientated under the assumption of perfect bonding between the GPL and the polymer matrix. This will result in the variations of the two Euler angles that identify the orientation of GPLs, i.e., θ to θ_s_ and ϕ to ϕ_s_. The coordinates of point A as shown in Figure 2 at the rim of the GPL disk in the global coordinate system before bi-axial stretching can be expressed as

(11) $$x_{1}^{\prime }=\frac{d_{\mathrm{GPL}}^{\prime }}{2}\sin\theta \cos\varphi ,\;x_{2}^{\prime }=\frac{d_{\mathrm{GPL}}^{\prime }}{2}\sin\theta \sin\varphi ,\;x_{3}^{\prime }=\frac{d_{\mathrm{GPL}}^{\prime }}{2}\cos\theta$$

and after stretching as

(12) $$x_{1}=\frac{d_{\mathrm{GPL}}}{2}\sin\theta_{s}\cos\varphi_{s},\;x_{2}=\frac{d_{\mathrm{GPL}}}{2}\sin\theta_{s}\sin\varphi_{s},\;x_{3}=\frac{d_{\mathrm{GPL}}}{2}\cos\theta_{s}$$

where $d_{\mathrm{GPL}}^{\prime }$ denotes the GPL diameter before stretching. Combining Equations (11) and (12), we have the following expressions

(13) $$\left\{ \begin{array}{l} \frac{x_{1}}{x_{1}^{\prime }}=\frac{d_{\mathrm{GPL}}\sin\theta_{s}\cos\varphi_{S}}{d_{\mathrm{GPL}}^{\prime }\sin\theta \cos\varphi }={\left[\left(1+\varepsilon_{3}\right)\left(1+\varepsilon_{2}\right)\right]}^{-\frac{\nu }{1-\nu }}\\ \frac{x_{2}}{x_{2}^{\prime }}=\frac{d_{\mathrm{GPL}}\sin\theta_{s}\sin\varphi_{S}}{d_{\mathrm{GPL}}^{\prime }\sin\theta \sin\varphi }=1+\varepsilon_{2}\\ \frac{x_{3}}{x_{3}^{\prime }}=\frac{d_{\mathrm{GPL}}\cos\theta_{s}}{d_{\mathrm{GPL}}^{\prime }\cos\theta }=1+\varepsilon_{3} \end{array}\right.$$

Neglecting the elongation of the GPL as discussed, the Euler angles after stretching can be derived in terms of the two original Euler angles from Equation (13) as

(14) $$\left\{ \begin{array}{l} \tan\theta_{S}=\frac{1+\varepsilon_{2}}{1+\varepsilon_{3}}\cdot \frac{\sin\varphi }{\sin\varphi_{s}}\tan\theta \\ \tan\varphi_{S}={\left(1+\varepsilon_{3}\right)}^{\frac{\nu }{1-\nu }}{\left(1+\varepsilon_{2}\right)}^{\frac{1}{1-\nu }}\tan\varphi \end{array}\right.$$

Equation (14) indicates that the filler in the unit cell is re-orientated and its Euler angles depend on bi-axial stretching strains. Therefore, the ODF required for micromechanics becomes a function of stretching strains instead of unity as that for composites with random and uniform distribution of reinforcing fillers. As mentioned previously, ODF is defined as the probability density of GPL orientation. For any distribution of fillers, it needs to satisfy [58,59]

(15) $$\rho \left(\varphi ,\theta \right)\geq 0\;\mathrm{and}\;\frac{1}{4\pi }\int_{0}^{2\pi }\int_{0}^{\pi }\rho \left(\varphi ,\theta \right)\sin\theta d\theta d\varphi =1$$

Assume a total number of *G* fillers are randomly and uniformly dispersed in the RVE before stretching (as shown in Figure 1). Then the number of fillers dispersed in the ranges (θ, θ + dθ) and (ϕ, ϕ + dϕ) in the RVE can be determined as [60]

(16) $$dN_{\begin{array}{l} \theta ,\theta +d\theta \\ \varphi ,\varphi +d\varphi \end{array}}=\frac{1}{4\pi }G\rho \left(\varphi ,\theta \right)\sin\theta d\theta d\varphi$$

After applying bi-axial stretching, these fillers within the ranges (θ, θ + dθ) and (ϕ, ϕ + dϕ) will be re-orientated to the ranges (θ_s_, θ_s_ + dθ_s_) and (ϕ_s_, ϕ_s_ + dϕ_s_). The same number of the fillers within the new ranges after stretching can be expressed as [61]

(17) $$dN_{\begin{array}{l} \theta_{s}{,\theta }_{s}{+d\theta }_{s}\\ \varphi_{s}{,\varphi }_{s}{+d\varphi }_{s} \end{array}}=\frac{1}{4\pi }G\rho (\varphi_{s},\theta_{s})\sin\theta_{s}{d\theta }_{s}{d\varphi }_{s}=dN_{\begin{array}{l} \theta ,\theta +d\theta \\ \varphi ,\varphi +d\varphi \end{array}}$$

where ρ(ϕ_s_, θ_s_) is the ODF after bi-axial stretching. Based on Equation (14), ϕ_s_, θ_s_, sin θ_s_, dθ_s_ and dϕ_s_ can be derived in terms of the original Euler angles ϕ and θ. Then the ODF after bi-axial stretching can be derived as

(18) $$\rho \left(\varphi_{s},\theta_{s}\right)=\frac{\frac{{\left(\frac{1+\varepsilon_{3}}{1+\varepsilon_{2}}\times \frac{{\sin\varphi }_{s}}{\sin\varphi }\right)}^{1/2}}{{\left(1+\varepsilon_{3}\right)}^{\frac{\nu }{1-\nu }}{\left(1+\varepsilon_{2}\right)}^{\frac{1}{1-\nu }}\cos^{2}\varphi_{s}+\frac{1}{{(1+\varepsilon_{3})}^{\frac{\nu }{1-\nu }}{\left(1+\varepsilon_{2}\right)}^{\frac{1}{1-\nu }}}\sin^{2}\varphi_{s}}}{{\left[{\left(\frac{1+\varepsilon_{3}}{1+\varepsilon_{2}}\right)}^{-1}\cdot {\left(\frac{{\sin\varphi }_{s}}{\sin\varphi }\right)}^{-1}\cos^{2}\theta_{s}+\frac{1+\varepsilon_{3}}{1+\varepsilon_{2}}\cdot \frac{{\sin\varphi }_{s}}{\sin\varphi }\sin^{2}\theta_{s}\right]}^{3/2}}$$

This obtained new ODF can be reduced to uni-axial stretching case when ε_1_ = ε_2_ = (1 + ε_3_)^−*ν*^ − 1. 

Equation (18) indicates that the ODF after stretching depends on the Poisson’s ratio of the composites, which relies on both the concentration and orientation of the GPL reinforcements. For random and uniform distribution of reinforcing fillers, rule of mixture can be used to approximate the Poisson’s ratio. However, to the best of our knowledge, no related work has been found on determining Poisson’s ratio with the consideration of the GPL concentration as well as the filler orientation. Regardless of the composition of the two-phase composites, it can be expected the Poisson’s ratio of the composites should be falling between the values of the GPL and the polymer. To simplify the modelling, the Poisson’s ratio of the GPL/polymer composites will be fixed as constant and typical values, i.e., *ν* = 0.1, 0.3 and 0.5, will be selected for the micromechanics model. 

**Figure 3:**
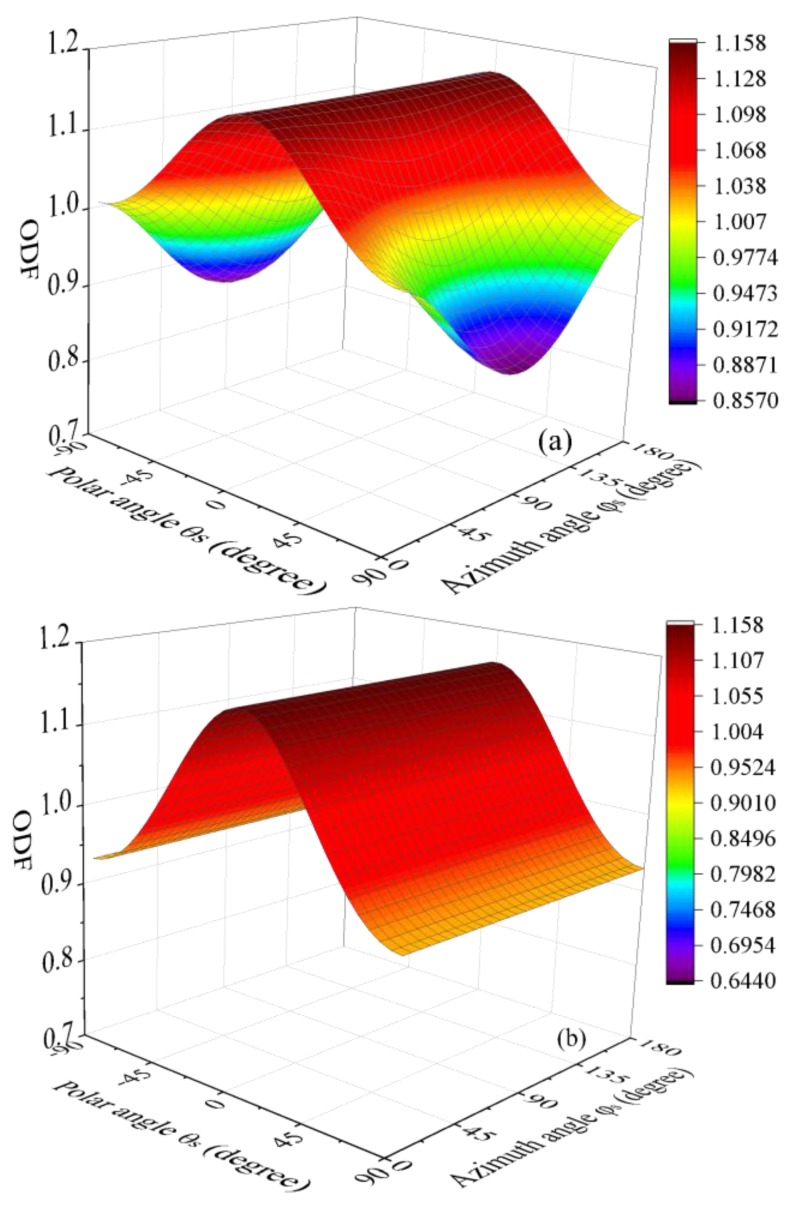

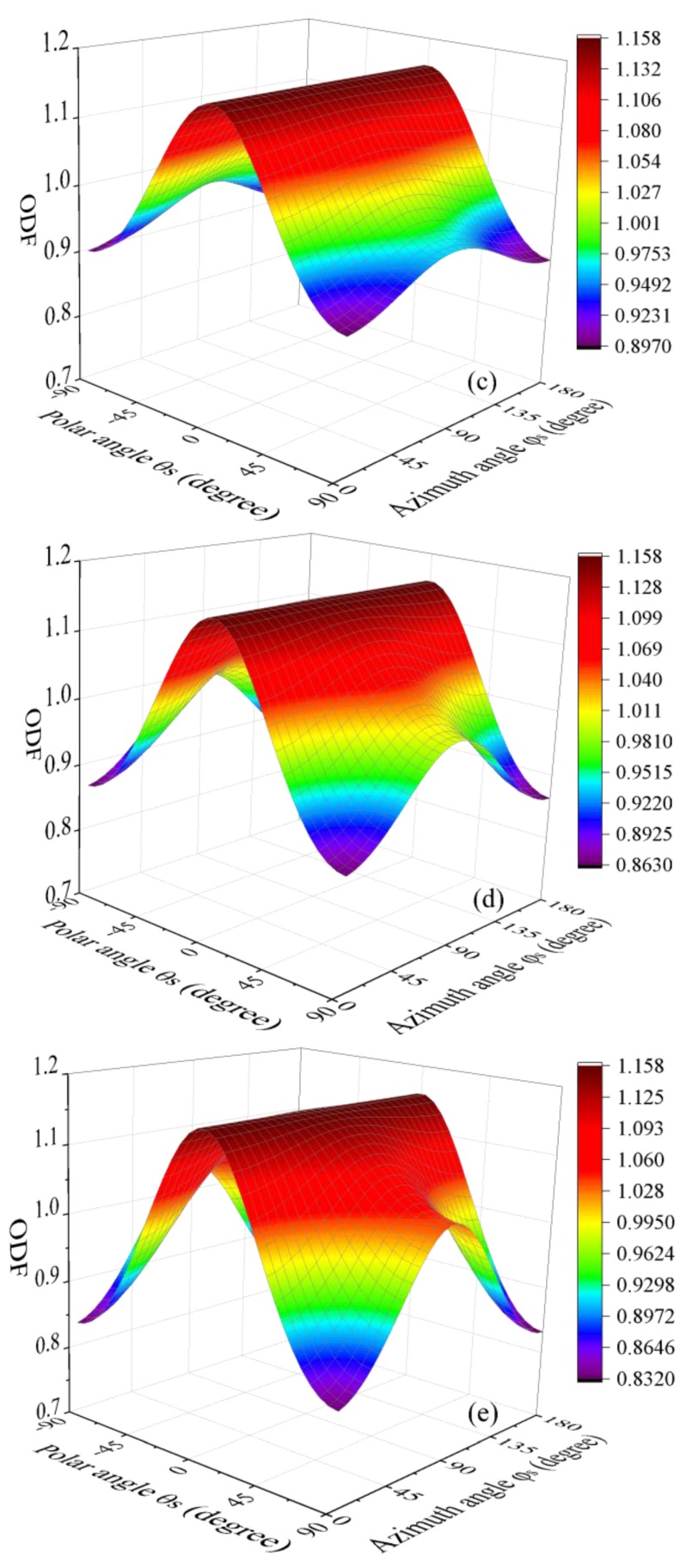

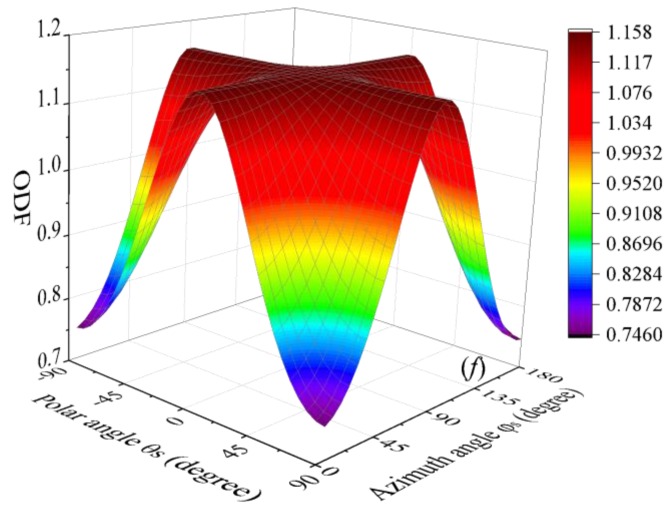
Orientation distribution function (**a**) ε_2_ = –ε_3_; (**b**) uni-axial stretching, ε_2_ = −0.48ε_3_; (**c**) ε_2_ = −0.25ε_3_; (**d**) ε_2_ = 0; (**e**) ε_2_ = 0.25ε_3_; (**f**) ε_2_ = ε_3_.

Figure 3 demonstrates the ODF after stretching with for different strain ratios. The strain in *X*_3_ direction and Poisson’s ratio are fixed as constants, i.e., ε_3_ = 5% and *ν* = 0.5, respectively. Here it should be noted that for most polymers, the Poisson’s ratio is normally smaller than 0.5. However, for composites with certain polymers as matrix, i.e., rubber, it is well-accepted to adopt 0.5 as the Poisson’s ratio due to their very limited compressibility. Taking the Poisson’s ratio as 0.5 in present work is for case study to eliminate the effects of volume expansion, which results in reduction in filler’s concentration. So we can focus on the study of the re-orientation effects. Therefore, unless stated otherwise, Poisson’s ratio will be chosen as 0.5 for parametric study thereafter. Obviously, GPLs tend to re-align along the principle stretching direction (θ_s_ = 0°) as indicated by the value of the ODF, which is larger than 1. The increase of the stretching strain in *X*_2_ direction enhances the re-alignment of GPLs in this direction (ϕ_s_ = 90°). Seen from Figure 3a, the ODF drops in *X*_2_ direction (ϕ_s_ = 90°) compared to the ODF for uni-axial stretching. This is because when ε_2_ = –ε_3_, the composite is compressed in *X*_2_ direction, which deteriorates the re-alignment of fillers along *X*_2_ direction.

**Figure 4:**
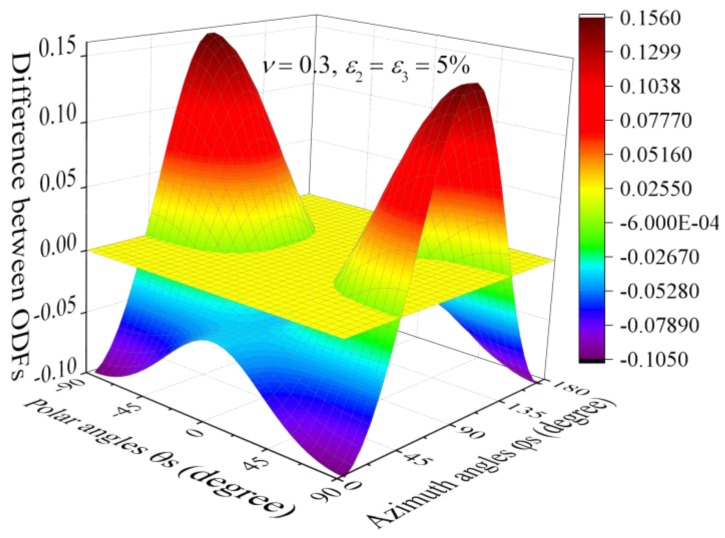
Difference between two ODFs.

The comparison in Figure 3 indicates that compared to uni-axial stretching, there exists critical Euler angles, for which the stretching in *X*_2_ direction does not change the ODF. For example, the contour graph in Figure 4 shows the difference between the two ODFs, i.e., ρ_bi-axial_ − ρ_uni-axial_, where ρ_uni-axial_ and ρ_bi-axial_ denote the ODFs under uni-axial stretching (ε_3_ = 5%) and equal bi-axial stretching (ε_3_ = ε_2_ = 5%), respectively. The flat plane represents the limiting case when ρ_bi-axial_ − ρ_uni-axial_ = 0. The two curves intersected by the contour and the plane result in the critical Euler angles. Obviously, bi-axial stretching increases the ODF above the flat plane while it decreases the ODF under the plane. 

**Figure 5:**
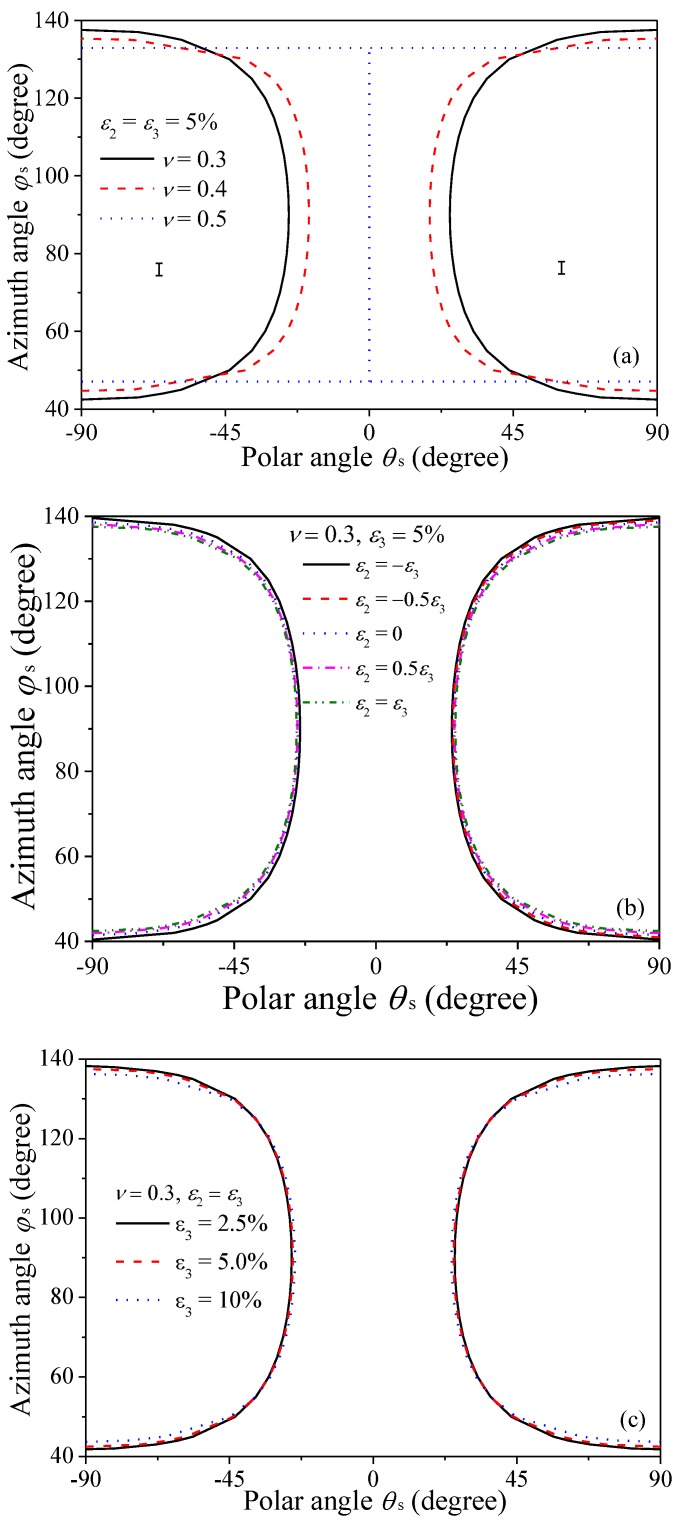
Critical Euler angles (**a**) Different Poisson’s ratios; (**b**) Different strain ratios; (**c**) Different stretching strains.

Figure 5 plots the shape of the critical Euler angles with considering the effects of Poisson’s ratio, strain ratio and stretching strain in *X*_3_ direction. The significant variations in the shape and area of region Ι in Figure 5a indicate that Poisson’s ratio has considerable effects on the critical Euler angles and Young’s modulus of the composites. With fixed Poisson’s ratio, Figure 5a,b suggests that the stretching strain ratio and the stretching strain in *X*_3_ direction have limited effects on the critical Euler angles. 

## 4. Results and Discussion

**Figure 6:**
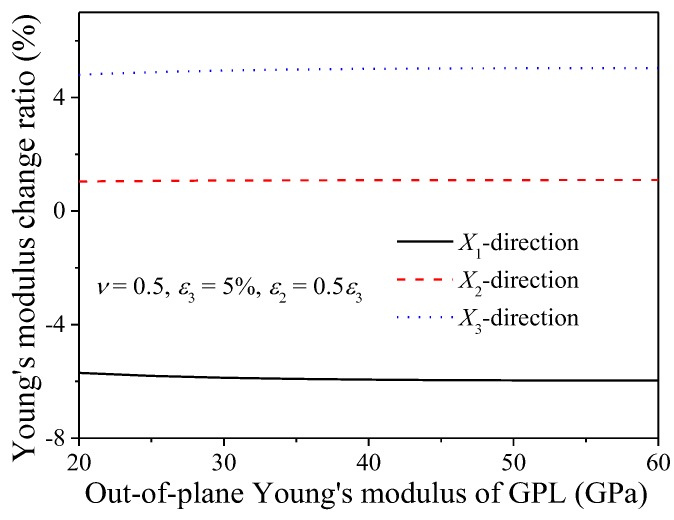
Effect of GPL out-of-plane Young’s modulus on Young’s modulus change ratio of GPL/epoxy nanocomposites.

To validate the micromechanics model, Young’s modulus predicted in present study will be compared to the experimental results by Wang et al. [49]. In Wang’s experiments, two grades of GPL, namely GnP-5 and GnP-C750, were used as the reinforcing fillers and epoxy 828 together with curing agent m-phenylene diamine (m-PDA) were selected as the constituents of the polymer matrix. The two grades of GPL have the same thickness, i.e., *t*_GPL_ = 5–10 nm, but they have different diameters and surface areas. The diameter and surface area of GnP-5 are 5 μm and 150 m^2^/g, respectively, while they are less than 1 μm and 750 m^2^/g, respectively, for GnP-C750. Utilizing various methods, including stirring, sonication and milling, the fabricated nanocomposite sample was observed to be a homogeneous mixture with well-distributed GPLs [49]. The mass densities of the epoxy and the GPL are ρ_M_ = 1.2 g/cm^3^ and ρ_GPL_ = 2.0 g/cm^3^ [49,50]. The Young’s modulus of the epoxy is *E*_M_ = 2.72 GPa and the in-plane Young’s modulus of the GPLs is *E*_in_ = 1.0 TPa. According to previous studies, the out-of-plane Young’s modulus of the GPL was predicted to be within 20–60 GPa [16], which was argued to be the modulus of exfoliation in the graphite *c*-axis (out-of-plane) [50,62]. Before validating the micromechanics modelling, Figure 6 investigates the effects of the out-of-plane Young’s modulus of GPL on the overall Young’s modulus of the composites, in which the Young’s modulus change ratio is defined as *η* = (*E*_CS_ − *E*_CO_)/*E*_CO_, where *E*_CO_ and *E*_CS_ are the Young’s moduli of the composites before and after stretching, respectively. It can be seen that Young’s modulus change ratios in the three directions are not sensitive to the out-of-plane Young’s modulus of GPLs, which agrees with previously reported observations [16,19,21]. The same trend can also be found for other Poisson’s ratios. Therefore, unless stated otherwise, the out-of-plane Young’s modulus of the composites will be chosen as *E*_ou_ = 40 GPa. 

**Figure 7:**
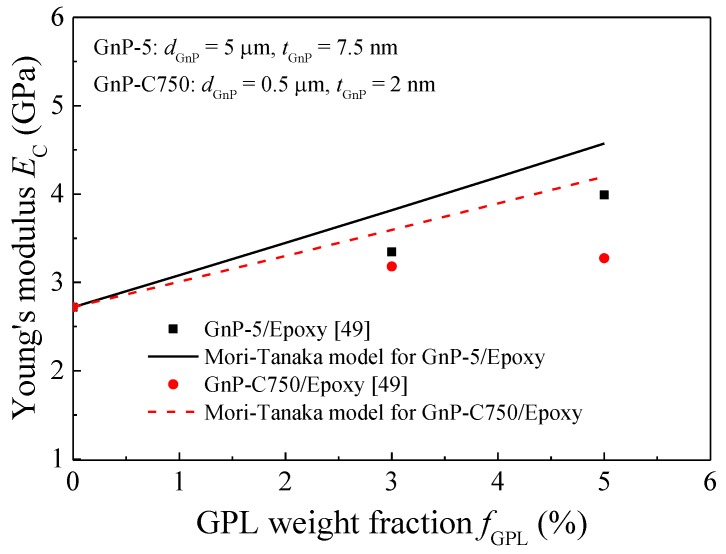
Comparison between present results and experimental data.

Figure 7 compares the present modelling results with experimental data. Seen from this figure, both theoretical and experimental results exhibit that GPLs with larger diameter-to-thickness ratio, *d*_GPL_/*t*_GPL_, have better reinforcing effect on the Young’s modulus of the composites. This phenomenon was explained by Wang et al. [49] that larger GPL diameter-to-thickness ratio facilitated better load transfer from the matrix to the GPL, leading to higher modulus. Compared to experimental results, the micromechanics model approximately overestimates the Young’s for both GnP-5 and GnP-C750 reinforced epoxy composites by 15–20%. Such overestimation can be attributed to the assumptions made for the micromechanical model. For example, GPLs were assumed as flat disks randomly and uniformly dispersed in the polymer matrix. However, curvature and agglomeration usually exist in GPLs due to their large diameter-to-thickness ratio and strong Van der Waals force among neighboring fillers, which were evidenced to deteriorate the mechanical properties of the composites. In addition, perfect bonding with no slipping between GPLs and the polymer matrix is assumed. However, from the perspective of manufacturing, the bonding between GPLs and the polymer matrix can be significantly weakened by unexpected bubbles and contamination, which obviously results in the decrease of the bonding strength and the load transfer between the reinforcing fillers and the polymer matrix. As indicated by Skountzos et al. [16], better interaction between graphene and polymer will result in more significantly improved mechanical properties. Therefore, the perfect bonding with neglection of interfacial effects will overestimate the Young’s modulus of the composites. It should be pointed out that in addition to the above comparison, more experimental results, especially the ones with considering stretching effects on Young’s modulus of the composites, should be used to further validate the model. However, this paper is the first attempt to quantitatively identify the effects of stretching induced reorientation of GPLs on the mechanical properties of the composites. To the best of the authors’ knowledge, no related experimental or theoretical results have been found on such effects. 

**Figure 8:**
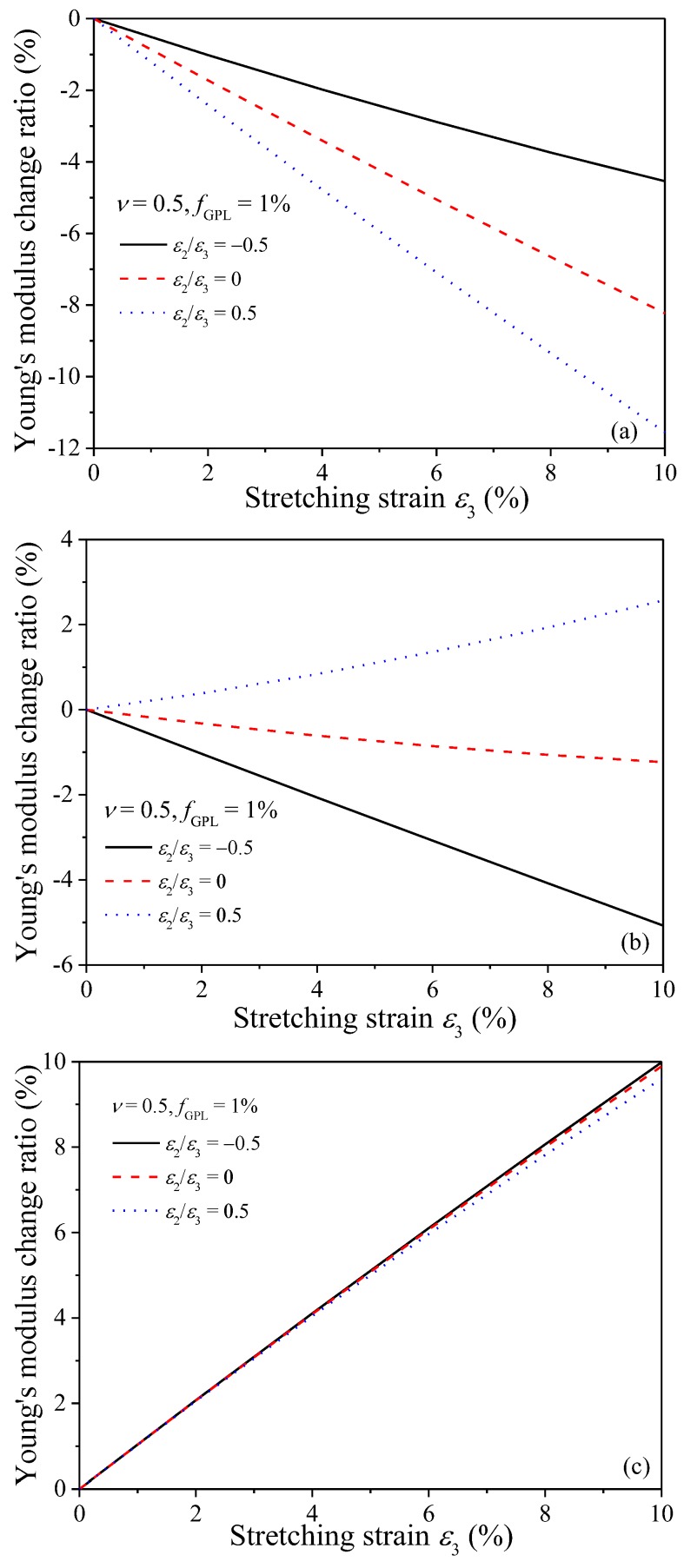
Variation of Young’s modulus change ratio of GPL/epoxy nanocomposites with stretching strain *ε*_3_ (**a**) *X*_1_-direction; (**b**) *X*_2_-direction; (**c**) *X*_3_-direction.

Figure 8 plots the variation of Young’s modulus change ratio with the stretching strain ε_3_. With fixed GPL weigh fraction, i.e., *f*_GPL_ = 1.0%, the increase of stretching strain in *X*_3_ direction significantly decreases/increases the Young’s moduli in *X*_1_/*X*_3_ directions, respectively, regardless of the strain ratio. For fixed stretching strain ε_3_, the Young’s modulus change ratio in *X*_1_ and *X*_3_ direction increases as the strain ratio increases. In addition, the Young’s modulus change ratio is less sensitive to the strain ratio in *X*_3_ direction than that in *X*_1_ direction. In contrast, the variation of Young’s modulus change ratio in *X*_2_ direction heavily depends on the strain ratio. For example, as the stretching strain *ε*_3_ increases, the Young’s modulus change ratio increases in *X*_2_ direction when ε_2_/ε_3_ = −0.5 and 0 while it increases when ε_2_/ε_3_ = 0.5. These phenomena can be explained by the fact the stretching enhances the re-alignment of GPL fillers in this direction while it deteriorates the re-alignment in the other two directions. Therefore, the re-alignment of GPL fillers in *X*_3_ and *X*_2_ directions due to the increased strains decreases the Young’s modulus change ratio in *X*_1_ direction. Figure 8b indicates that for smaller strain ratio, i.e., ε_2_/ε_3_ = −0.5 and 0, the decrease in Young’s modulus by the stretching strain ε_3_ in *X*_2_ direction dominates the variation of the Young’s modulus. However, for larger strain ratio, i.e., ε_2_/ε_3_ = 0.5, the increase in Young’s modulus by the stretching in *X*_2_ direction becomes the prominent. Apparently, Figure 8c advises the stretching in *X*_3_ direction always governs the variation of the Young’s modulus in this direction.

**Figure 9:**
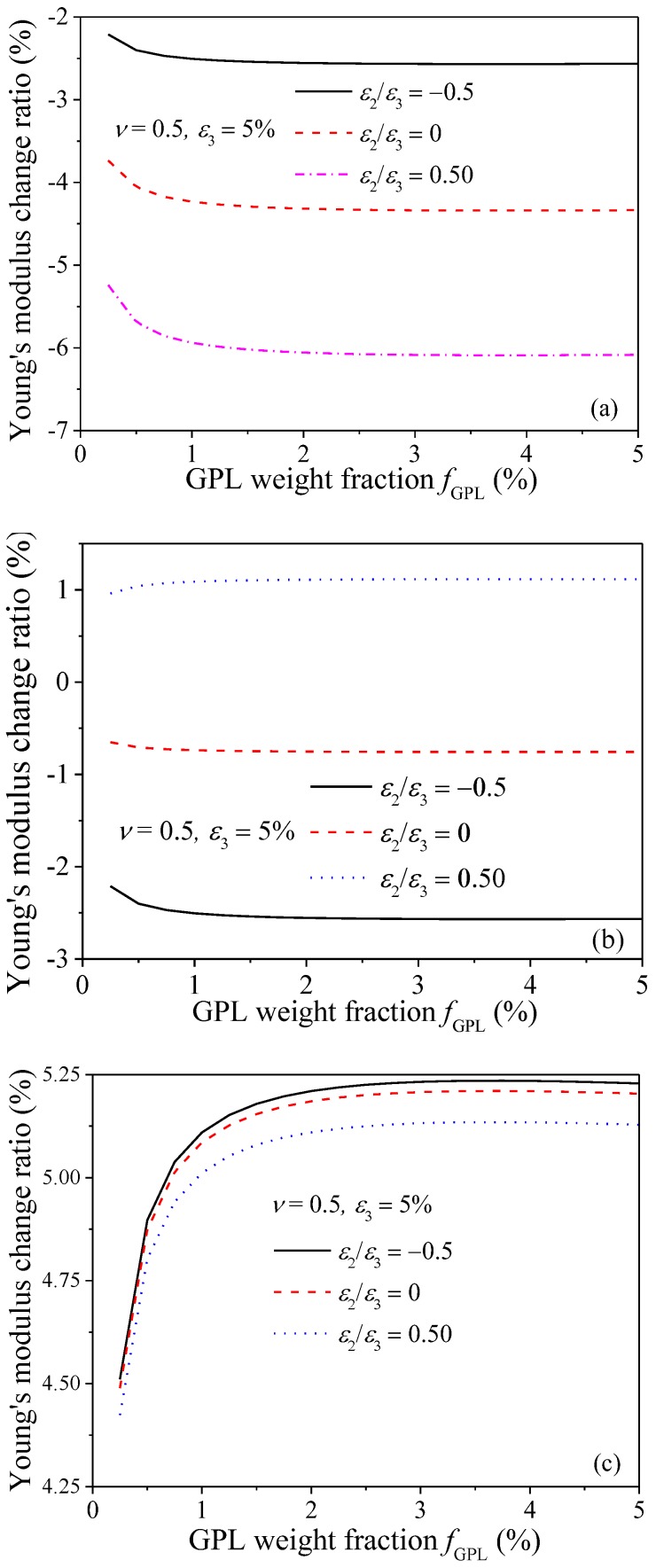
Variation of Young’s modulus change ratio of GPL/epoxy nanocomposites with GPL weight fraction (**a**) *X*_1_-direction; (b) *X*_2_-direction; (**c**) *X*_3_-direction.

Figure 9 shows the variation of the Young’s modulus change ratio with GPL weight fraction *f*_GPL_ for nanocomposites subjected to different stretching strain ratios, where the stretching strain in *X*_3_ direction is fixed as a constant, i.e., ε_3_ = 5%. When the GPL/epoxy nanocomposites are subjected to stretching, the variation of the Young’s modulus change ratio is sensitive to smaller *f*_GPL_, especially in *X*_1_ and *X*_3_ directions. As *f*_GPL_ increases to sufficiently large, the variation of Young’s modulus change ratio becomes negligible. This can be explained by the fact that for composites with lower filler concentration, a limited number of GPLs exist in the matrix, any small changes in the GPL distribution, i.e., the re-alignment along the stretching direction, will affects the magnitude of the Young’s modulus significantly. In contrast, a larger number of GPLs are dispersed in polymer matrix with higher filler concentration and any change to the fillers will have relatively limited effects on the composites. The comparisons among the three figures indicate that increase of the stretching strain in *X*_2_ direction increases the Young’s modulus in *X*_2_ direction while it decreases the Young’s modulus in *X*_1_ and *X*_3_ direction, which agrees with the observations in Figure 8. 

**Figure 10:**
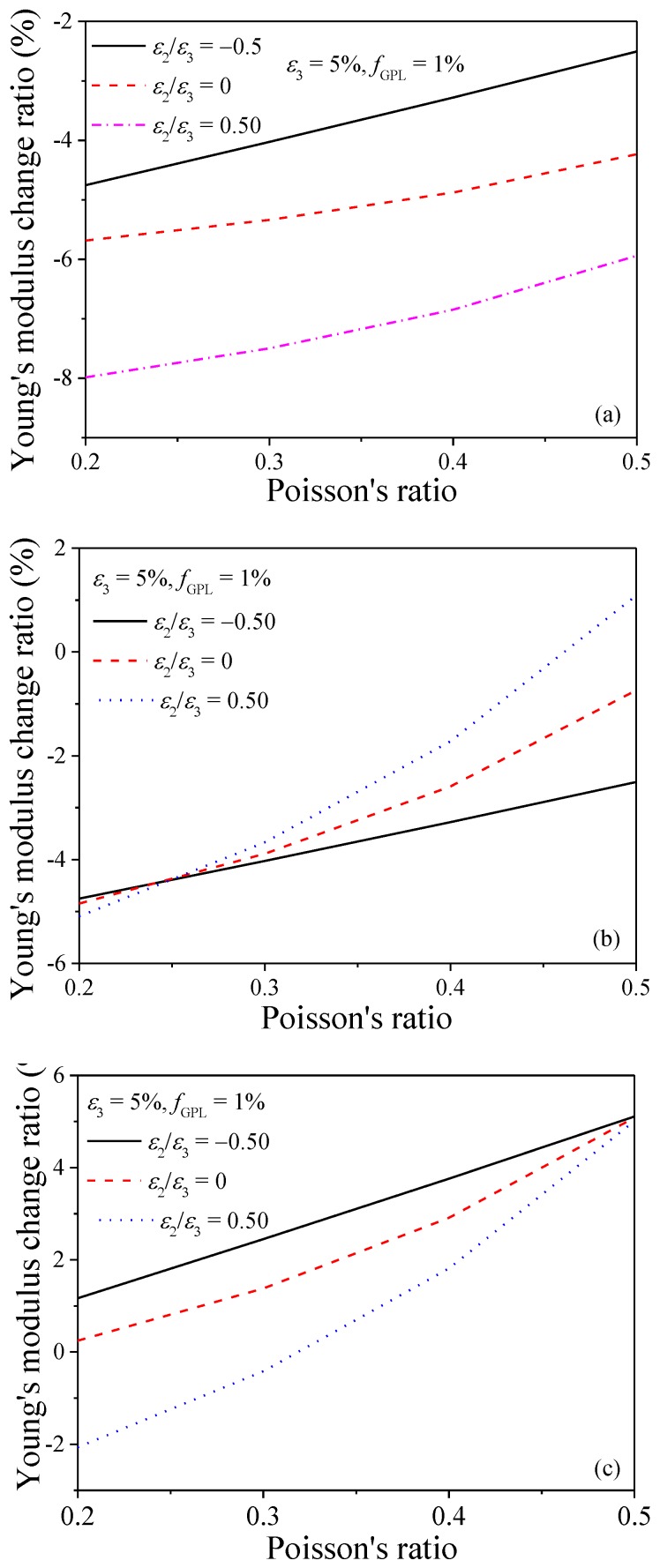
Effect of Poisson’s ratio on Young’s modulus change ratio of GPL/epoxy nanocomposites (**a**) *X*_1_-direction; (**b**) *X*_2_-direction; (**c**) *X*_3_-direction.

Figure 10 investigates the effects of Poisson’s ratio on the Young’s modulus change ratio of the GPL/epoxy nanocomposites when subjected to stretching. As indicated in Figure 5, larger Poisson’s ratio is beneficial for the re-alignment of GPLs in stretching directions. Thus, it is easily understood that the Young’s modulus change ratio increases in the two stretching directions (as shown in Figure 10b,c). The improvement of re-alignment of GPLs in *X*_2_ and *X*_3_ directions due to larger Poisson’s ratio is expected to decrease the Young’s modulus in *X*_1_ direction. However, Figure 10a shows the Young’s modulus increases with the increase of the Poisson’s ratio. This is because larger Poisson’s ratio induces less volume expansion when nanocomposites are stretched, resulting in higher effective concentration and Young’s modulus. Thus, the increase of the Young’s modulus in *X*_1_ direction suggests that the Poisson’s ratio induced volume expansion effects dominate the variation of the Young’s modulus over the effects of GPL re-alignment in *X*_1_ direction. 

**Figure 11:**
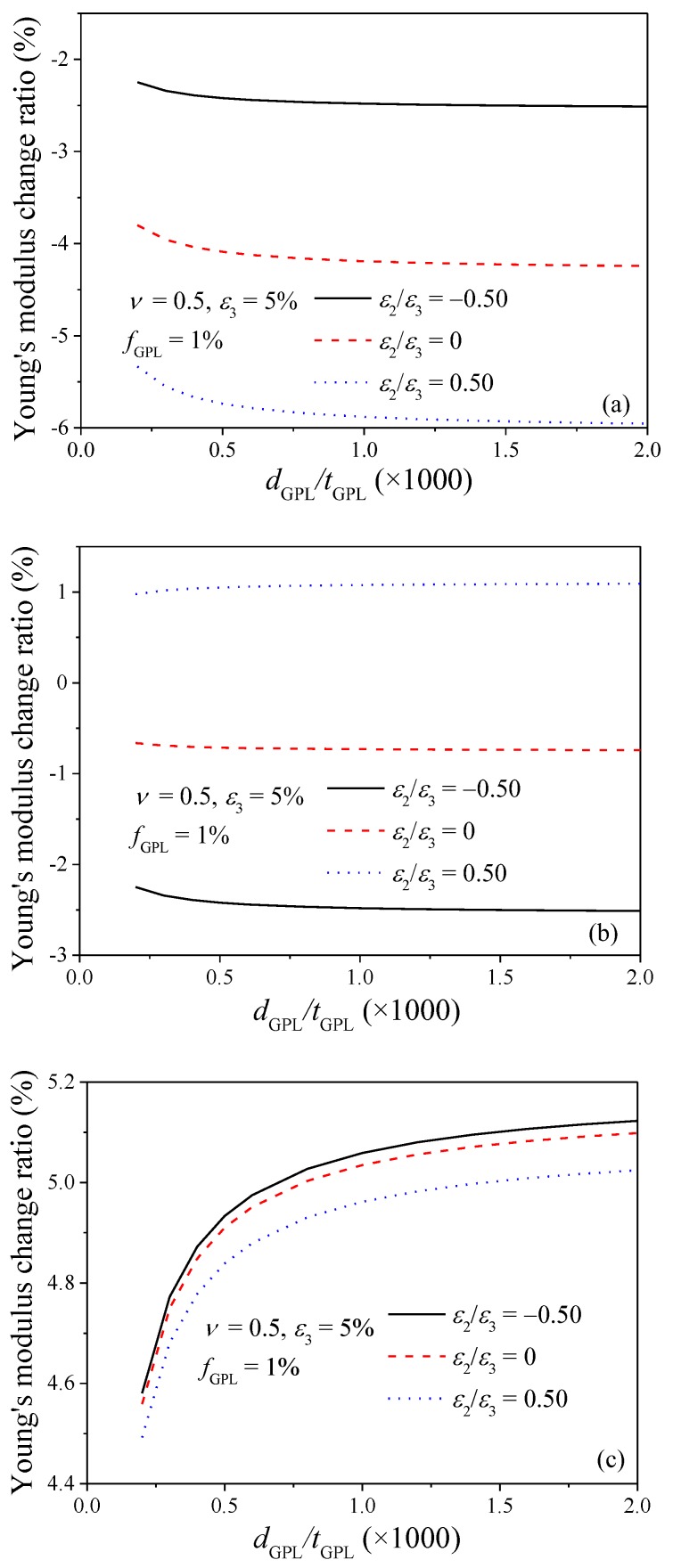
Effect of GPL dimension on Young’s modulus change ratio of GPL/epoxy nanocomposites (**a**) *X*_1_-direction; (**b**) *X*_2_-direction; (**c**) *X*_3_-direction.

The effects of the GPL geometry on the Young’s modulus of GPL/epoxy composites are presented in Figure 11. The diameter of the GPL is fixed as a constant while its thickness varies. From the figures, it can be seen that the Young’s modulus in *X*_2_ direction is less sensitive to GPL diameter-to-thickness ratio *d*_GPL_/*t*_GPL_ compared to the variations in *X*_1_ and *X*_3_ directions. The Young’s modulus decreases and increases significantly in *X*_1_ and *X*_3_ directions, respectively, when the diameter-to-thickness ratio is relatively small, i.e., *d*_GPL_/*t*_GPL_ < 500. This indicates that GPLs with larger diameter-to-thickness ratio have better reinforcing effects on the Young’s modulus of the nanocomposites in the principle stretching direction. This agrees with the findings reported by Liu et al. [12]. Again, similar trend of the effect of stretching strain in *X*_2_ direction on the Young’s modulus of the composites in the three directions is observed. 

## 5. Conclusions

The re-orientation effects of GPLs induced by bi-axial stretching on the Young’s modulus of the GPL/polymer composites are investigated by Mori-Tanaka micromechanics model, where the GPL orientation distribution is characterized by ODF. It is found that GPLs tend to re-align along stretching directions. The modelling results demonstrate that the out-of-plane Young’s modulus of the GPL has limited effect on the overall Young’s modulus of the composites. Higher Poisson’s ratio is beneficial for the re-alignment of the GPL fillers along stretching direction, resulting in the increase of Young’s modulus. Compared to uni-axial stretching, the stretching in *X*_2_ direction increases the Young’s modulus in this direction while it decreases the Young’s modulus in *X*_3_ direction. It is observed that GPLs with larger diameter-to-thickness ratio are better reinforcing fillers. The study on the effects of bi-axial stretching on the Young’s modulus will provide guidelines for the design and prediction of the mechanical properties when mechanical stretching is adopted as method to re-align GPL fillers.
